# The Hoosier cavefish, a new and endangered species (Amblyopsidae, *Amblyopsis*) from the caves of southern Indiana

**DOI:** 10.3897/zookeys.412.7245

**Published:** 2014-05-29

**Authors:** Prosanta Chakrabarty, Jacques A. Prejean, Matthew L. Niemiller

**Affiliations:** 1 Museum of Natural Science, Ichthyology Section, 119 Foster Hall, Department of Biological Sciences, Louisiana State University, Baton Rouge, Louisiana 70803, USA; 2 University of Kentucky, Department of Biology, 200 Thomas Hunt Morgan Building, Lexington, KY 40506, USA

**Keywords:** Cryptic diversity, GenSeq, new species, subterranean, taxonomy

## Abstract

We describe a new species of amblyopsid cavefish (Percopsiformes: Amblyopsidae) in the genus *Amblyopsis* from subterranean habitats of southern Indiana, USA. The Hoosier Cavefish, *Amblyopsis hoosieri* **sp. n.**, is distinguished from *A. spelaea*, its only congener, based on genetic, geographic, and morphological evidence. Several morphological features distinguish the new species, including a much plumper, Bibendum-like wrinkled body with rounded fins, and the absence of a premature stop codon in the gene *rhodopsin*. This is the first new cavefish species described from the United States in 40 years and exemplifies how molecular data can alert us to the presence of otherwise cryptic biodiversity.

## Introduction

The teleost family Amblyopsidae (order Percopsiformes) comprises North America’s largest clade of stygobiotic (obligate cave-dwelling) fishes and has long been of interest to evolutionary biologists and ecologists studying adaptations to extreme subterranean habitats (reviewed in Niemiller and Poulson 2010). The taxonomy of Amblyopsidae has remained relative stable since the 1950s when Woods and Inger (1957) published a major taxonomic revision of the family, which included assigning *Troglichthys rosae* (Eigenmann 1898) to the genus *Amblyopsis*, recognizing *Forbesichthys papilliferus* (Forbes, 1882) as a junior synonym of *Forbesichthys agassizii* (Putnam, 1872), and recognizing *Typhlichthys eigenmanni* Charlton, 1933, *Typhlichthys wyandotte* Eigenmann, 1905 and *Typhlichthys osborni* Eigenmann, 1905 as junior synonyms of *Typhlichthys subterraneus* Girard, 1859. Cooper and Kuehne (1974) described the most recent amblyopsid to be recognized, *Speoplatyrhinus poulsoni*, a stygobiotic species known only from a single cave system in northern Alabama.

There has been a revival in amblyopsid systematics and taxonomy in recent years, as several studies have examined higher-level phylogenetic relationships as well as population level differentiation using molecular approaches (Niemiller and Fitzpatrick 2008, Dillman et al. 2011, Niemiller et al. 2012, 2013a, d). Several significant systematic revisions have been proposed, including the assignment of *Amblyopsis rosae* back into the genus *Troglichthys* (Niemiller et al. 2013a), resurrection of *Forbesichthys papilliferus* (Niemiller et al. 2013a) and resurrection of *Typhlichthys eigenmanni* (Niemiller et al. 2012) based on biogeographical and phylogenetic evidence. Eight species are currently recognized in Amblyopsidae, including three surface or primarily spring-dwelling taxa: *Chologaster cornuta* Agassiz 1853, *Forbesichthys agassizii*, and *Forbesichthys papilliferus*; and five obligate cave-dwelling taxa: *Typhlichthys subterraneus*, *Typhlichthys eigenmanni*, *Troglichthys rosae*, *Speoplatyrhinus poulsoni* and *Amblyopsis spelaea* DeKay 1842 (sensu Niemiller et al. 2013a, d). However, the multilocus phylogenetic study by Niemiller et al. (2012) also uncovered substantial cryptic genetic variation associated with hydrological boundaries in *Typhlichthys subterraneus*, suggesting that biodiversity is underestimated in *Typhlichthys* and perhaps other cavefish lineages. The recognition of such cryptic species has important implications for conservation and management (e.g., Niemiller and Fitzpatrick 2008, Niemiller et al. 2013b), but also for comparative ecological and evolutionary studies (e.g., Niemiller et al. 2013a, c).

**Figure 1.**
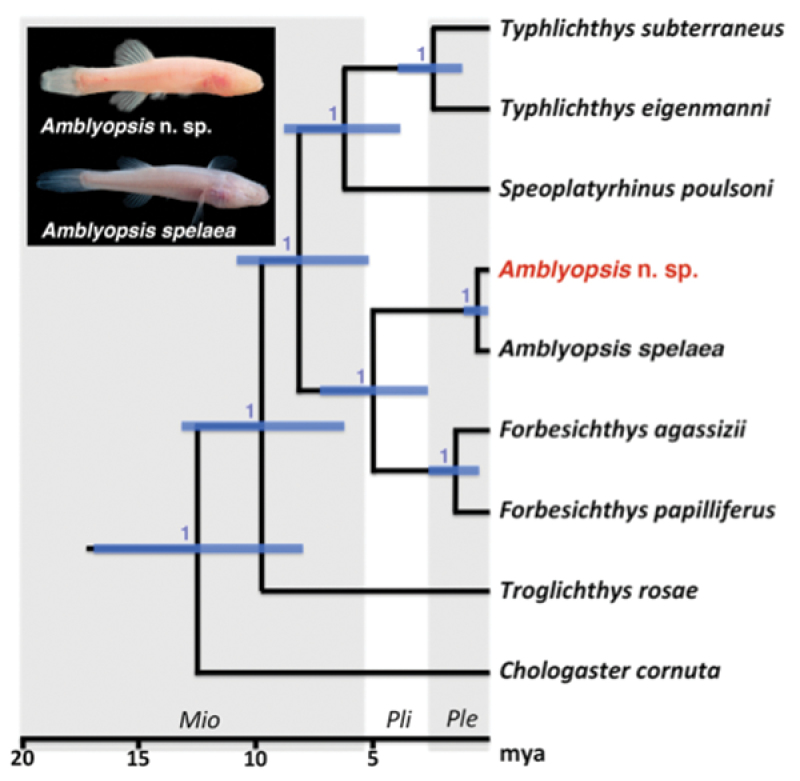
Phylogeny of Amblyopsidae. Modified from Niemiller et al. (2013d), showing a northern lineage of *Amblyopsis* found north of the Ohio River in Indiana (members of the new species described herein) and a southern lineage in Kentucky (*Amblyopsis spelaea* sensu n.).

A recent study of *Amblyopsis spelaea* based on phylogeographic structure of one mitochondrial and four nuclear loci identified two evolutionary lineages associated with the modern Ohio River (Niemiller et al. 2013d): a northern lineage located north of the Ohio River in Indiana and a southern lineage in Kentucky. Niemiller et al. (2013d) suggested that these two lineages might warrant recognition as distinct species (Fig. 1).

These northern and southern lineages were recovered as reciprocally monophyletic for two of the nuclear loci, *s7* and *rhodopsin*, and the mtDNA locus *nd2*. Little variation was exhibited at the nuclear loci *rag1* and *tbr.* The gene tree estimated from the mitochondrial *nd2* locus contained two strongly supported clades (Bayesian posterior probability > 0.95) separated by 27 mutational steps with observed average uncorrected pairwise genetic distances of 3.1% between these lineages. Observed uncorrected pairwise genetic distances were considerably lower for all nuclear loci; however, segregating variation was observed in *s7* and *rhodopsin*. A single nucleotide substitution at *s7* segregated between the northern and southern lineages, whereas three nucleotide substitutions segregated at *rhodopsin*. The differences in *rhodopsin* include a mutation that results in a premature stop codon in the open reading frame of all individuals sampled in the southern lineage that is absent from the northern lineage.

**Figure 2.**
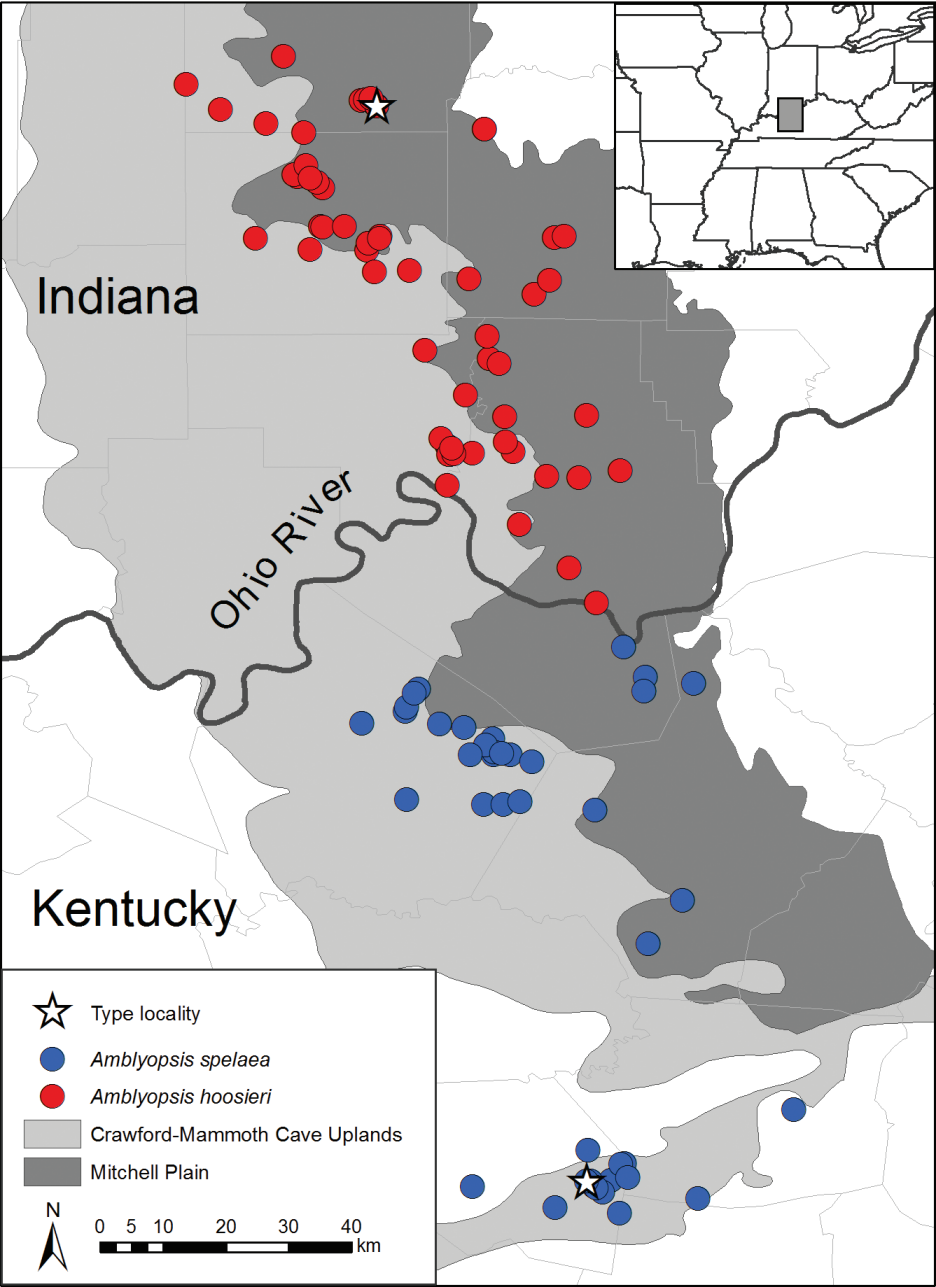
Distribution of *Amblyopsis* spp., *Amblyopsis spelaea* and *Amblyopsis hoosieri*, in the Mitchell Plain and Crawford-Mammoth Uplands of Indiana and Kentucky.

The results of Niemiller et al. (2013d) strongly implicate the Ohio River as a significant barrier to dispersal and, consequently, an isolating mechanism facilitating divergence between populations located north and south of the river (Fig. 2). Poulson (1960) examined variation in morphology throughout much the northern distribution of *Amblyopsis spelaea* and found subtle differences in pigmentation and rudimentary eye size; however, he only examined specimens from the Mammoth Cave region for the southern range of the species. Therefore, it is unclear whether phenotypic differences exist between phylogenetic lineages identified by Niemiller et al. (2013d). In this study, we examined morphological variation from individuals of the northern and southern lineages of *Amblyopsis* identified by Niemiller et al. (2013d), and included specimens from populations for which material for DNA sequencing were previously unavailable. Based on our results, we describe the northern lineage as *Amblyopsis hoosieri* sp. n., from subterranean waters of southern Indiana.

## Materials and methods

Institutional abbreviations are as follows: CAS (California Academy of Science); INHS (Illinois Natural History Survey); IU (Indiana University); LSUMZ (Louisiana State University Museum of Natural Science); UMMZ (University of Michigan Museum of Zoology), YPM (Yale Peabody Museum). Non-type materials examined include: *Amblyopsis spelaea*: INHS 50129 (n=1), 60573 (n=1); UMMZ 146991 (n=1); 179149 (n=1); YPM 25294 (n=8, n=1 cleared and stained). Materials examined for the new species are listed below as types. Cleared and stained specimens were prepared following modifications of the method outlined by Taylor and Van Dyke (1985). Specimens were stained in Alcian blue for two days, then bleached in a potassium hydroxide solution, neutralized in a hydrogen peroxide + potassium hydroxide mixture, then transferred into trypsin to clear. Specimens were then placed in Alizarin red for 20 minutes and subsequently transferred to distilled water for 24 hours. Specimens were then placed back into trypsin for a final clearing and slowly moved up to full glycerin using a staggered glycerin/potassium hydroxide mixture that slowly increased glycerin in 10% intervals up to 100% over a period of a week.

Radiographs were made for all specimens using a Faxitron x-ray cabinet. All meristics (numbers of fin rays and vertebrae) were counted using these radiographs.

Morphometric measurements, following Hubbs and Lagler (2004), were recorded to the nearest 0.1 mm using digital calipers and included: standard length (SL), head length (HL), head width, upper jaw length, body depth (depth of body at deepest point), pectoral-fin length, caudal-fin length, pelvic-fin length, dorsal-fin base, anal-fin base, caudal peduncle length, caudal peduncle width, caudal peduncle depth, predorsal length, prepelvic length, and preanal length. Other traditional measurements, such as, snout length, interorbital width and orbit diameter are excluded because of the absence of eyes (externally) in these taxa. An additional measurement, body width, was taken directly posterior to the opercula on the widest part of the body. We also conducted an analysis of covariance (ANCOVA) to compare body width and body depth among species with standard length as a covariate in R (v3.0.2; R Development Core Team 2013).

## Results

**Figure 3.**
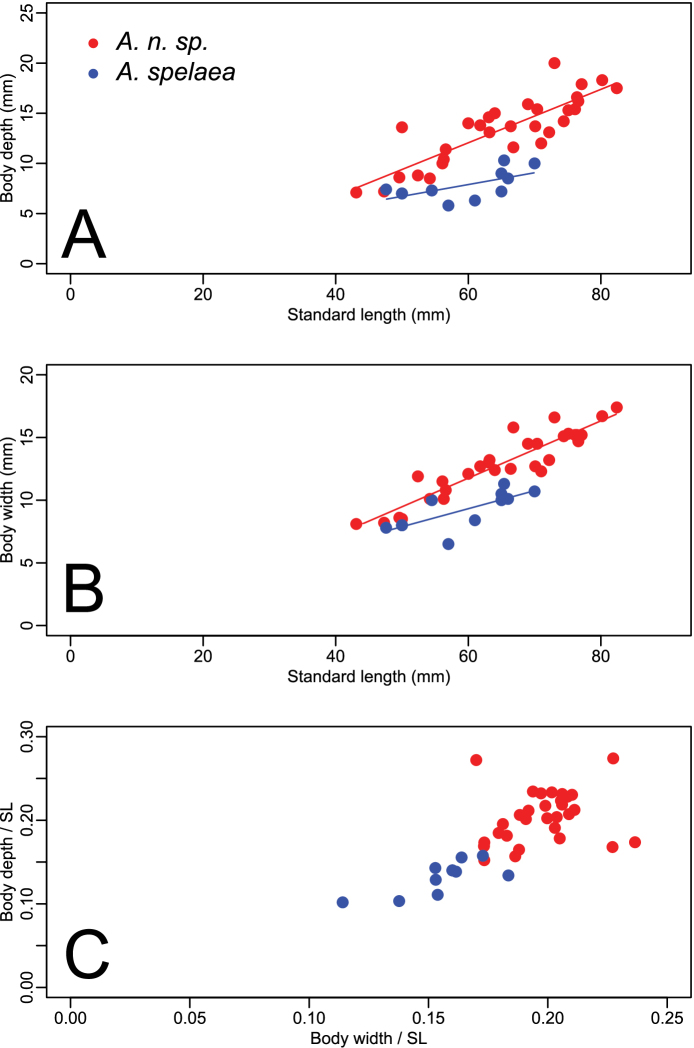
Plots illustrating the relationships between body depth, body width and standard length (SL). Circles in red represent specimens of *Amblyopsis hoosieri*, sp. n., from north of the Ohio River in Indiana; triangles in blue represent *Amblyopsis spelaea* from south of the Ohio River in Kentucky. (**a**) body depth versus standard length, (**b**) body width versus standard length, and (**c**) and body depth versus body width as proportions of standard length.

Forty-one specimens were examined, 30 from north of the Ohio River in Indiana, and 11 from south of the river in Kentucky. Specimens from Kentucky included the type locality of *Amblyopsis spelaea*, Mammoth Cave in Edmonson County. Figure 3 (A–C) shows the results of analyses of covariance (ANCOVA) comparing body depth and body width versus SL. Notably, *Amblyopsis hoosieri* has a deeper body compared to *Amblyopsis spelaea* (ANCOVA, p < 0.001; Fig. 3A, C). The body is also wider in *Amblyopsis hoosieri* compared to *Amblyopsis spelaea* (ANCOVA, p < 0.001; Fig. 3B, C). Except for juveniles (those under 50 mm), individuals of the new species are deeper and wider bodied than individuals of *Amblyopsis spelaea* (Fig. 3C). Given individuals of the same standard length, one would expect those of the new species to be much more robust.

## Systematic accounts

### Amblyopsis hoosieri

Niemiller, Prejean & Chakrabarty sp. n.

http://zoobank.org/688BC3D5-7773-41E8-961F-67E3E4902BE2

http://species-id.net/wiki/Amblyopsis_hoosieri

**Figure 4.**
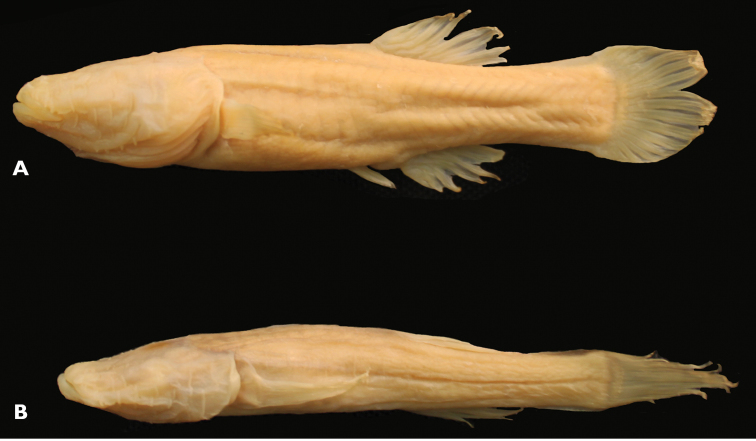
Comparative image of two similarly sized individuals of both species of *Amblyopisis*. *Amblyopsis hoosieri*, holotype, INHS 106675, 75.1 mm SL, Bronson’s Cave, Lawrence Co., Indiana (**A**); a specimen of *Amblyopsis spelaea* (YPM ICH 25294) of similar SL (67 mm SL) showing the more elongate and sculpted (versus plump) body, pointed fins, less prominent myomeres and more prominent papillae on the body (**B**).

**Figure 5.**
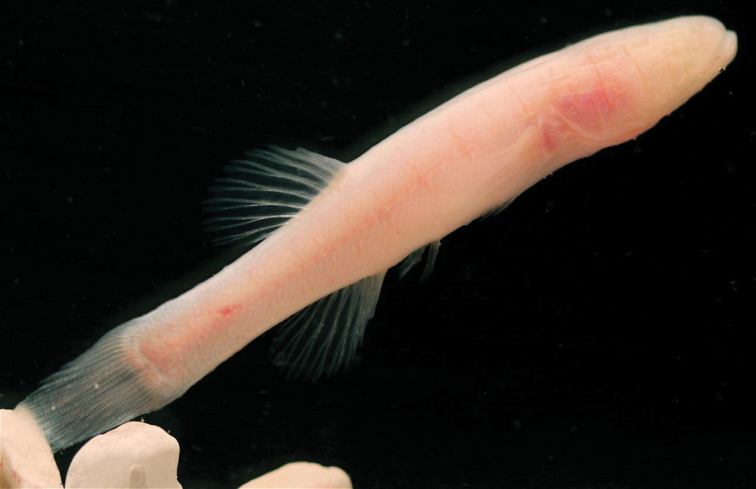
Photograph of a paratype of *Amblyopsis hoosieri* in life, YPM ICH 25304, 60.7 mm SL. Photograph by M.L. Niemiller.

Figures 4A , 5 [Fig F6] –7 ; Table 1

Amblyopsis spelaea “N” Niemiller et al. 2013d: pg. 9 (Fig. 3)
Amblyopsis spelaea : Simon 2011: pg. 230–231; Fig. 146 (in part)
Amblyopsis spelaea : Poulson 1963: pg. 267, 269 Upper Twin, Spring Mill (in part)
Amblyopsis spelaea : Woods and Inger 1957: pg. 241, 243–245; Fig. 5 (in part)

#### Type material.

**Holotype.** INHS 106675, Bronson’s Cave (White River Dr.) Spring Mill State Park, Lawrence County, Indiana, USA; 38°44', -86°25'; 9 December 1962, W.U. Bringham [formerly in lot 102504]

**Paratypes.** INHS 40424 (n=12 in ETOH; n=2 cleared and stained), Bronson’s Cave (White River Dr.) Spring Mill State Park, Lawrence County, Indiana, 5 April 1964, W.U. Brigham, G.W. Barlow & J. Mertz; INHS 60574 (n=1), Spring, (Lost River Dr.) Near West Baden, Orange County, Indiana, 18 January 1904, N.H. Haden; INHS 102504 (n=4), same data as holotype; LSUMZ 17419 (n=1), same as UMMZ 157175, formerly of that lot; LSUMZ 17420 (n=1), same data as UMMZ 65000, formerly of that lot; UMMZ 65000 (n=2), Twin Caves, near Mitchell, Lawrence County, Indiana, 17 May 1924, Hubbs & party; UMMZ 90379 (n=2), Sibert’s Well Cave, beside Wyandotte Cave, Indiana, 17 August 1930, P. Hickie; UMMZ 113550 (n=1), Lost River, Indiana, ca. 2 mi NE of Orangeville, September 1935, J.J. & W.P. Petravicz; UMMZ 114890 (n=2), Donaldson’s Cave, Spring Mills State Park, Lawrence County, 16 June 1934, A.E. Emerson; UMMZ 144604 (n=1), Stream in Sibert’s Well Cave, Wyandotte, Crawford County, Indiana, 01 October 1942, L. Hubricht; UMMZ 146992 (n=1), Stream in Sheep Cave, near Wyandotte, Crawford County, Indiana, 01 September 1939,, L. Hubricht; UMMZ 146994 (n=3), Stream in Bronson Cave, Spring Mill State Park, Lawrence County, Indiana, 2 September 1939, L Hubricht; UMMZ 157174 (n=1), “possibly” Donaldson farm caves near Indiana University, Indiana, C.H. Eigenmann; UMMZ 157175 (n=1), same as previous lot; UMMZ 157176 (n=1), same as previous lot; UMMZ 160944 (n=1), Twin Cave, Mitchell, Lawrence County, Indiana, 18 June 1924, F.N. Blanchard; YPM 25304 (n=2), Donaldson Cave, Spring Mill State Park, Lawrence County, Indiana, 21 December 2007, M.L. Niemiller et al.; YPM 25305 (n=1), Blue Springs Caverns, Lawrence County, Indiana, 21 December 2007, M.L. Niemiller et al. [Note tissue samples and published sequences are available from paratypes in YPM 25304 and 25305 (Niemiller et al. 2013d) and these correspond to genseq-2 following the nomenclature of Chakrabarty et al. (2013). GenBank numbers for these sequences from the paratypes are reported in Table 2.]

**Table 1.** Measurements and meristic counts of species of *Amblyopsis*. Standard length is in mm. Other measurements are percentages of standard length or head length. Values reported are means and ranges are in parentheses. For meristic counts, number of specimens with a count value is in parentheses.

Character	*Amblyopsis hoosieri* (Indiana)	*Amblyopsis spelaea* (Kentucky)
	N=30	N=11
Standard length	65.2 (43.1–82.4)	59.2 (47.6–70.0)
	*Percentage of standard length*	
Head length	34.1 (30.6–37.0)	33.0 (30.0–37.2)
Body depth	20.3 (15.2–27.2)	13.2 (10–16.6)
Body width	19.7 (17–23.7)	15.5 (11.4–18.3)
Pectoral-fin length	18.2 (11.9–24.0)	24.0 (20.0–27.5)
Pelvic-fin length	8.0 (5.6–9.7)	8.4 (5.7–10.4)
Caudal-fin length	19.9 (12.0–25.3)	25.5 (19.1–29.4)
Dorsal-fin base	12.0 (9.5–15.8)	11.0 (7.5–12.8)
Anal-fin base	10.8 (6.5–14.9)	11.6 (6.6–14.0)
Caudal-peduncle length	29.8 (27.0–34.7)	30.0 (27.7–33.2)
Caudal-peduncle depth	11.0 (9.4–12.9)	9.7 (8.6–11.0)
Caudal-peduncle width	6.1 (4.7–8.3)	5.9 (4.9–6.7)
Predorsal length	59.0 (50.6–64.1)	60.3 (58.5–63.0)
Preanal length	63.4 (58.6–67.2)	59.6 (56.0–63.1)
Prepelvic length	55.3 (50.5–59.7)	51.8 (48.6–55.5)
	*Percentage of head length*	
Head width	70.0 (51.5–82.4)	65.0 (56.3–70.4)
Upper jaw length	32.8 (27.2–37.5)	33.4 (30.0–40.0)
Counts		
Dorsal-fin rays	11(1), 10(16), 9(11), 8(2)	10(3), 9(8)
Anal-fin rays	10(7), 9(21), 8(2)	10(3), 9(8)
Vertebral Count	30(16), 29(14)	31(1), 30(9), 29(1)

**Table 2.** GenBank accession numbers for sequences from *Amblyopsis hoosieri* paratypes, which receive genseq-2 ranking in the classification of Chakrabarty et al. (2013). Sequences were used and derived from the study of Niemiller et al. (2013d). Genes are listed in columns: *tbr1* (T-box brain), *rag1* (recombinating activating protein 1), *s7* intron 1 (ribosomal protein s7, intron 1), *nd2* (NADH dehydrogenase subunit 2), *rhod* (rhodopsin).

Gene	*tbr1*	*rag1*	*s7*	*nd2*	*rhod*
Specimen #					
**YPM 25304.A**	JX978106	JX978036	JX977966	JX977896	JX459497
**YPM 25304.B**	JX978107	JX978037	JX977967	JX977897	JX459498
**YPM 25305**	JX978108	JX978038	JX977968	JX977898	JX459499

#### Morphological diagnosis.

**Figure 6. F6:**
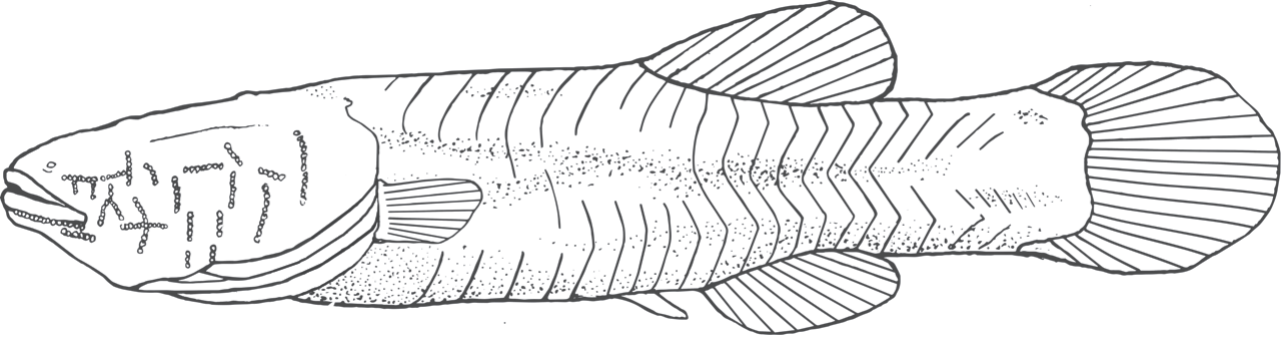
Illustration of *Amblyopsis hoosieri* based on the holotype, INHS 106675, 75.1 mm SL. Illustration by Nathan Coussou.

*Amblyopsis hoosieri* can be distinguished from its only congener, *Amblyopsis spelaea*, by having a more plump, fleshy and rounded body (versus sculpted and thin) with Bibendum-like wrinkles along myomeres (versus tight skin) and by having rounder pectoral fins (versus pointed; Figs 4–6). Additionally, the mechansensory papillae on the body and caudal fin are reduced in size and less elevated on the skin (versus conspicuous in *Amblyopsis spelaea*).

#### Molecular diagnosis.

Average uncorrected pairwise genetic distance at the mitochondrial NADH dehydrogenase 2 (*nd2*) locus between *Amblyopsis hoosieri* and *Amblyopsis spelaea* is 3.1%, with 27 mutations separating the two species. *Amblyopsis hoosieri* and *Amblyopsis spelaea* can also be readily diagnosed using molecular data at the nuclear *rhodopsin* gene, a G-coupled photoreceptor expressed in the retina of the vertebrate eye. All *rhodopsin* sequences of *Amblyopsis hoosieri* code for the amino acid glutamine (Q) at position 184, whereas *Amblyopsis spelaea* possesses a point mutation that results in a premature stop codon at this position. In addition, *Amblyopsis hoosieri rhodopsin* codes for the amino acid valine (V) at position 254, whereas *Amblyopsis spelaea* codes for the amino acid phenylalanine (F). A single mutation in intron 1 of ribosomal protein S7 (*s7*) also distinguishes the two species.

#### Description.

**Figure 7.**
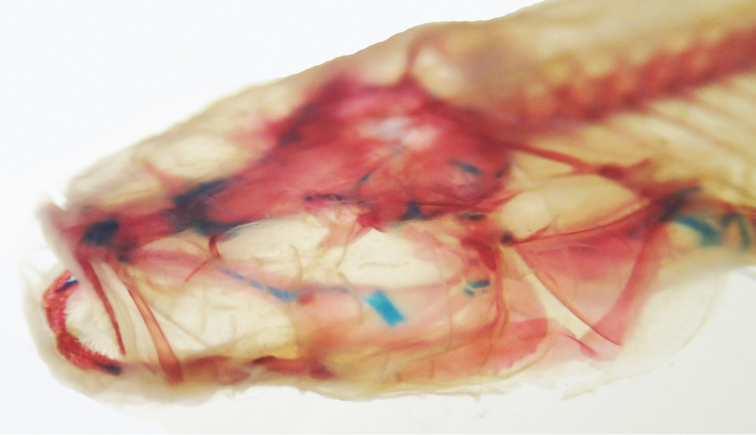
Cleared and stained image of the head of *Amblyopsis hoosieri*. Note lack of eye or a clearly defined bony orbit. Specimen is a paratype from INHS 40424, 71.3 mm SL.

Robust, blind (eye not developed, Fig. 7), unpigmented cavefish typically reaching between 60–80 mm in adult standard length. Head large (about ¼ body length) flat dorsally but broad; head widest part of body. Body widest at operculum, narrows to caudal fin. Body rectangular, dorsal and vertical profile of body nearly symmetrical; deepest point at dorsal-fin origin. Fleshy protuberance present anterior to dorsal-fin origin; similar protuberance at anal-fin origin. Body narrows posterior to dorsal- and anal-fin origins, narrowest point at midpoint of caudal peduncle. Body plump, wrinkly in appearance (as in Bibendum) prominent deep myomeres present. Deep groove on ventral side of body from operculum and anus to pelvic fin. Scales inconspicuous and cycloid.

Superficial mechanosensory neuromasts on papillae (Eigenmann 1909, Niemiller and Poulson 2010) present in rows of 5–30 on head. Papillae concentrated at mouth; fewer posteriorly on head. Most rows of papillae vertical, far fewer horizontal. Horizontal rows typically connect two to four other vertical rows. Most mechanosensory papillae on dorsal side of head concentrated and aligned posterior to, or between, nares. No mechanosensory papillae in central area of dorsal region of head. Papillae present dorsal to braincase in two horizontal rows. No lateral line on body. Mechanosensory papillae on body much smaller than those on head and aligned in vertical rows; some scattered papillae near dorsal-fin base. Inconspicuous papillae present on caudal fin in horizontal rows of two or three in both dorsal and ventral half of fin, vertical row present at base.

Anterior nares small, tube shaped; posterior nares slightly larger with small anterior flap, but otherwise circular. Lips somewhat thin and fleshy. Lower jaw slightly longer than upper jaw.

Vertical through dorsal-fin origin between more anterior pelvic-fin origin and more posterior anal-fin origin. Anal-fin and dorsal-fin insertions near same vertical plane. All fins relatively short and rounded. Anus located anteriorly on body, behind isthmus of united gill membranes (i.e., jugular). Caudal skeleton upturned and asymmetrical (externally appearing homocercal), with last half centrum (preural 1 + ural 1) including hypural (3-X; following Rosen 1962) and entirely associated with dorsal half of caudal fin. Five or six principal caudal-fin rays supported by each hypural plate (ventral hypural 1+2; again following Rosen 1962).

Branchiostegals six in number, robust and prominent. Papilliform flap at dorsal origin of operculum. Six or seven gill rakers on ceratobranchial of first gill arch. Rakers short, stubby and denticulated. Central and upper tooth plates also heavily denticulated.

Buccal teeth villiform, in three to five rows. Individual teeth unicuspid, slender and long; teeth deeply embedded in mouth so only top 1/3 visible. Teeth recumbent, particularly those on upper jaw. Palatine and vomerine teeth also present.

Body uniformly depigmented, including inside mouth. Body pinkish-white, reddish near gills, fins transparent. In alcohol, body color uniformly yellowish/beige, fins opaque yellow.

#### Etymology.

The specific epithet *hoosieri* is in reference to this species being from the state of Indiana. It is also a reference to Indiana University, where biologist Carl H. Eigenmann was a Professor of Zoology and studied blind cave vertebrates, including populations of *Amblyopsis hoosieri* in Lawrence County just to the south of Bloomington (Eigenmann 1909). Indiana University was also home to the Father of American Ichthyology, David Starr Jordan, for most of his illustrious career. We derive the specific epithet from the proper noun “Hoosier.” Notably, the senior author of the manuscript is a fervent fan of Indiana Hoosier basketball while the first author is an alumni of the University of Michigan and is not. Suggested common name, Hoosier Cavefish.

#### Distribution.

*Amblyopsis hoosieri* occurs in caves developed in carbonate rock of the Crawford-Mammoth Cave Uplands and Mitchell Plain in the South-Central karst region of Indiana (Fig. 2) within the area that remained ice free throughout the Pleistocene Epoch (Woods and Inger 1957, Frushour 2012). Caves within the distribution of *Amblyopsis hoosieri* are primarily developed in Mississippian-aged limestones and carbonates belonging to the St. Louis and St. Genevieve Limestone (Frushour 2012). The northernmost locality occurs 16 km from the glacial maxima of the Illinoian glaciation. The distribution of *Amblyopsis hoosieri* is bounded to the north by the East Fork White River and the south by the Ohio River. The species has been documented from at least 74 localities in Crawford, Harrison, Lawrence, Orange and Washington counties, including 68 cave systems and six springs (Keith 1988, Pearson and Boston 1995, Lewis 2002a, Niemiller and Poulson 2010, Niemiller et al. 2013d). *Amblyopsis hoosieri* is known from the Lower White, Lower East Fork White, Patoka and Blue-Sinking watersheds.

#### Habitat.

*Amblyopsis hoosieri* is found primarily in larger cave streams at or near the water table where it has been observed in pools with low flow at depths as shallow as 0.1 m to > 2 m deep. *Amblyopsis* cavefishes from Indiana have been found in association with silt-sand, gravel, cobble and bedrock substrates (Poulson 1963, Pearson and Boston 1995, Niemiller and Poulson 2010). A preference for larger pools with relatively deep, slow-moving water with large breakdown boulders has been noted (McCandless 2005). During high flow conditions, cavefish seek refuge under ledges, in crevices or in areas of breakdown (Niemiller and Poulson 2010). These habitats and preferences are similar to those found in *Amblyopsis spelaea* (reviewed in Niemiller and Poulson 2010).

#### Life history.

Poulson (1960, 1963) provided the most significant study on the ecology of the species described herein as *Amblyopsis hoosieri* based primarily on cave populations near Mitchell in Lawrence County, Indiana, which is reviewed in Niemiller and Poulson (2010). *Amblyopsis hoosieri* has a well-defined annual reproductive cycle (Poulson 1963, Niemiller and Poulson 2010). Breeding presumably occurs during high water levels from February through April. Females brood eggs in their branchial cavities until hatching and continue to care for fry until yolk reserves are depleted 4–5 months later (Eigenmann 1909, Niemiller and Poulson 2010). Fry appear in late summer into early autumn. Growth rates are estimated at 1.0 mm month^-1^ but decline with age (Niemiller and Poulson 2010). Sexually maturity is likely reached in 3–4 years (Poulson 1963). Longevity is unknown but estimated to be at least 12–15 years (but perhaps 20+ years) based on growth rates and scale formation (Louis 1999, Niemiller and Poulson 2010). Documented prey of *Amblyopsis hoosieri* includes copepods, isopods, and amphipods. Larger individuals will feed on small crayfish (Poulson 1963, Niemiller and Poulson 2010). Predators have not been documented in nature and it’s thought that individuals of *Amblyopsis* are one of the top predators in cave systems they inhabit (Niemiller and Poulson 2010).

## Discussion

We describe a new species of North American cavefish, *Amblyopsis hoosieri*, that is distinguished from its sole congener *Amblyopsis spelaea* based on body and pectoral-fin shape and the absence of a stop codon in *rhodopsin* among other molecular and morphological features. In addition, the distributions of the two species of *Amblyopsis* are separated by the Ohio River, which has downcut through major cave-bearing rock strata and has subsequently isolated populations on the north (*Amblyopsis hoosieri*) and south (*Amblyopsis spelaea*) sides of the river. Cavefish diversity is certainly underestimated globally but perhaps particularly in North America: this is the first new cavefish species from the U.S. in 40 years.

Notably, previous cavefish researchers did not recognize this taxon as novel. Eigenmann (1899) noted that some individuals of *Amblyopsis* had more developed cones than others and Poulson (1960) noted qualitatively greater non-external pigmentation and degenerate “eye” size of specimens of *Amblyopsis spelaea* from south of the Ohio River. Unfortunately, due to the limited number of specimens available we were not able to fully examine these internal features. However, we found that there are several lines of evidence to distinguish the two species based on external morphological features, molecular data and geography.

Eigenmann (1905) described *Typhlichthys wyandotte* from “north of the Ohio River, from a well near Corydon, Indiana.” The type locality is located within the distribution of *Amblyopsis hoosieri* and well outside the known distribution of *Typhlichthys* in the Interior Plateau, which ranges from northern Alabama and northwestern Georgia through central Tennessee into south-central Kentucky (Mammoth Cave region). The type locality was believed to be destroyed (Woods and Inger 1957), although a well-like entrance into a cave has been located in Corydon, Indiana, and may be the type locality (Black, pers. comm. in Lewis 2002b). Unfortunately, the only known specimen (the holotype; formerly IU 4646, currently CAS 91988) is very badly damaged and in several pieces, with most of the head lost. In his description, Eigenmann (1905) stated that this species is more slender than *Typhlichthys* from south of the Ohio River. This condition is opposite of the situation in *Amblyopsis*, where individuals north of Ohio River are less slender than those south of the river. A survey of 200+ caves in the same drainage basin as the possible type locality has only documented *Amblyopsis* and has failed to find *Typhlichthys* (Lewis 1998). *Typhlichthys wyandotte* is currently considered a junior synonym of *Typhlichthys subterraneus* (Woods and Inger 1957), but it is unclear whether the poorly preserved holotype is a member of *Typhlichthys* or *Amblyopsis*.

Molecular data have become an important tool to help identify cryptic or otherwise poorly recognized species level diversity, particularly among subterranean taxa (Chakrabarty 2010, Chakrabarty et al. 2012, Sparks and Chakrabarty 2012). Generally lacking eyes, pigmentation, and other common features of sighted organisms, subterranean fish species have few diagnostic features. Molecular data have been used to discover and diagnose cavefish diversity only recently, but these data are powerful and can surely help increase our understanding of this poorly studied fauna.

**Conservation status.** *Amblyopsis spelaea*, and *Amblyopsis hoosieri* by extension, is considered endangered in Indiana because of presumed vulnerability to groundwater pollution and other perturbations of aquatic subterranean habitats. The species is considered “Endangered” (S1) in Indiana by NatureServe (2013) because of the few occurrences of occurrences, small population sizes and being restricted to subterranean habitats that are highly vulnerable to anthropogenic activities. *Amblyopsis spelaea* is considered “Vulnerable” on the IUCN Red List (Gimenez Dixon 1996). *Amblyopsis hoosieri* should have the same threat category at minimum or be at greater risk of extinction. *Amblyopsis hoosieri* is known from at least 74 localities, but most localities appear to represent sink rather source populations (Pearson and Boston 1995, McCandless 2005). However, a few cave systems contain large populations based on direct counts during visual encounter surveys that likely are source populations, including Eric’s River Cave in Crawford Co., and Blue Spring Caverns, Donaldson Cave and Upper Twin Cave, Lawrence Co. (Pearson and Boston 1995, McCandless 2005, Niemiller and Poulson 2010).

Potential threats to populations of *Amblyopsis hoosieri* are discussed in detail (for *Amblyopsis spelaea*) by Keith (1988), Pearson and Boston (1995), Lewis (2002a) and Niemiller and Poulson (2010). These threats include sedimentation related to agriculture, increased human visitation and collection, and groundwater pollution, particularly from pesticide, herbicide and fertilizer use. Some localities have been directly impacted by anthropogenic activities. Keith (1988) reported that two blind cavefish localities in Indiana were either partially or completely destroyed by quarrying. Groundwater contamination from pesticides was attributed to the cause of “broken-back syndrome” in the population at Donaldson Cave, Lawrence County (Keith and Poulson 1981). At least two populations are indirectly affected by commercial cave tours in Lawrence County (Pearson and Boston 1995; Niemiller and Poulson 2010). Over-collection for scientific studies during the late 1800s and early 1900s may have impacted some populations in Lawrence County. Dozens to hundreds of cavefish were collected from the “Mitchell Caves” (Bronson-Donaldson and Twin Cave systems) by Eigenmann, Payne and others (e.g., Eigenmann 1899, 1903, 1909; Ramsey 1901, Payne 1907) for experiments on cave adaptation. These caves are now protected and located within Spring Mill State Park. The state of Indiana has implemented measures to help protect populations of *Amblyopsis*, including restricting access to caves and regulating recreational activities permitted. However, the delineating of drainage basins and potential sources of contamination as well as protection of surface and subsurface drainage basins is probably the most important conservation measure to protect the species (Niemiller and Poulson 2010).

## Supplementary Material

XML Treatment for Amblyopsis hoosieri
